# A Case of Fatal Herpes Simplex Virus 1 Encephalitis Complicated by Status Epilepticus: From Elective Phlebectomy to Intensive Care

**DOI:** 10.7759/cureus.83346

**Published:** 2025-05-02

**Authors:** Ronnie Napoles, Sania L Siddiqui, Zaen U Manzoor, Sann Htoo

**Affiliations:** 1 Internal Medicine, HCA Florida Aventura Hospital, Aventura, USA; 2 Internal Medicine, Nova Southeastern University Dr. Kiran C. Patel College of Allopathic Medicine, Fort Lauderdale, USA; 3 Medicine, Nova Southeastern University Dr. Kiran C. Patel College of Allopathic Medicine, Fort Lauderdale, USA

**Keywords:** herpes simplex virus 1 (hsv-1), hsv-1, hsv-1 encephalitis, hsv-1 infection, status epilepticus

## Abstract

Herpes simplex encephalitis is most commonly caused by herpes simplex virus type 1 (HSV-1) and is believed to result from retrograde transport along the trigeminal and olfactory nerves into the central nervous system. This case report presents a 73-year-old female who arrived at the emergency department following the acute onset of confusion before an elective outpatient procedure. Her initial physical examination and diagnostic workup were unremarkable. By day six of hospitalization, the patient became progressively obtunded, with significant clinical deterioration marked by a Glasgow Coma Scale score of 5 and recurrent episodes of high-grade fever. Magnetic resonance imaging revealed inflammation and hyperintensities in the right frontotemporal lobes, highly suggestive of HSV encephalitis, and cerebrospinal fluid analysis confirmed HSV-1 infection. This case report discusses the rapid clinical progression of HSV-1 encephalitis, highlights potential complications, and reviews the acute management of a patient with a poor prognosis.

## Introduction

Herpes simplex encephalitis is a rare neurological disorder characterized by inflammation of the brain parenchyma, particularly the mesial temporal lobes [1]. Approximately 90% of cases are linked to herpes simplex virus type 1 (HSV-1), while type 2 (HSV-2) accounts for fewer than 10% [2]. These viruses, part of the herpesvirus family, contain double-stranded DNA and establish latency in neurons following initial infection of epithelial cells [2]. Host immune response and viral characteristics influence the severity and progression of the disease.

Although 60-90% of adults are seropositive for HSV-1, herpes simplex encephalitis remains rare, with 2-4 cases per million people annually [2]. The condition affects all age groups but is more common in young children and older adults, demonstrating a bimodal distribution with peaks in those under three years and over 50 years of age, without a gender predilection [1,2]. In contrast, HSV-2 encephalitis primarily affects neonates in the first three weeks of life [2].

Early symptoms of HSV-1 encephalitis, including fever, headache, nausea, and malaise, are often non-specific and can mimic respiratory or systemic infections [1,2]. Differential diagnoses include other viral encephalitides, post-infectious processes, brain neoplasms, and autoimmune or paraneoplastic encephalitis [3]. As the disease progresses, patients may develop prolonged altered mental status, seizures, focal neurological deficits, and behavioral or cognitive changes [2]. In one study of 106 individuals with herpes simplex encephalitis, hospital admissions were most often prompted by seizures (32%), abnormal behavior (23%), confusion or disorientation (13%), and loss of consciousness (13%) [4]. Diagnosis may be even more challenging in immunocompromised patients, who often exhibit minimal early neurological symptoms [2].

We report a case of HSV-1 encephalitis in an elderly patient who developed acute neurological symptoms before an elective outpatient procedure. This case highlights the unusual timing of symptom onset and the severe progression to status epilepticus, emphasizing the importance of early recognition and management of HSV-1 encephalitis.

This article was previously presented as a meeting abstract at the 2024 Eastern Pulmonary Conference (EPC) on September 12, 2024.

## Case presentation

A 73-year-old female with a past medical history of hyperlipidemia, vascular insufficiency, thrombophlebitis, and ovarian teratoma status post-oophorectomy and hysterectomy presented for an elective right saphenous vein phlebectomy. Before the procedure, the patient developed a sudden episode of confusion, lightheadedness, and shortness of breath and was transferred to the emergency department. Vital signs were within normal limits (see Table 1). On the physical examination, she was alert, awake, and oriented, and appeared non-toxic without focal neurological deficits. Her initial blood work revealed a hemoglobin of 12.2 g/dL, white blood cell count (WBC) of 5.2 × 10^3^/µL, platelets of 137 × 10^3^/µL, and elevated creatinine kinase of 208 U/L with a negative infectious workup (see Table 2). Baseline electrocardiogram showed normal sinus rhythm at 77 beats per minute with sinus arrhythmia. On preliminary workup, computed tomography (CT) angiography head showed an incidental 3 × 3 mm wide neck aneurysm in the distal right M1 segment of the middle cerebral artery. The patient was subsequently admitted for further evaluation.

**Table 1:** Initial vitals.

	Patient’s value	Reference values
Blood pressure	120/84 mmHg	<120/80 mmHg
Temperature	36.4°C	36.5–37.3°C
Respiration rate	17 respirations/minute	12–20 respirations/minute
Pulse	83 beats/minute	60–100 beats/minute
Pulse oximetry	100%	≥95%

**Table 2:** Initial laboratory tests.

Initial laboratory tests	Results	Reference values
White blood cell count	5.2 × 10^3^/µL	4.0–10.5 × 10^3^/µL
Hemoglobin	12.2 g/dL	11.2–15.7 g/dL
Platelet	137 × 10^3^/µL	150–400 × 10^3^/µL
Glucose	122 mg/dL	70–110 mg/dL
Blood urea nitrogen	24 mg/dL	6–22 mg/dL
Creatinine	0.50 mg/dL	0.43–1.13 mg/dL
Sodium	138 mmol/L	135–145 mmol/L
Potassium	4.9 mmol/L	3.5–5.2 mmol/L
Chloride	103 mmol/L	95–110 mmol/L
Carbon dioxide	27 mmol/L	19–34 mmol/L
Calcium	8.7 mg/dL	8.4–10.2 mg/dL
Alanine aminotransferase	21 U/L	10–60 U/L
Aspartate aminotransferase	42 U/L	10–40 U/L
Alkaline phosphatase	58 U/L	20–130 U/L
Total protein	7.4 g/dL	5.5–8.7 g/dL
Albumin	4.1 g/dL	3.2–5.0 g/dL
Bilirubin total	0.7 mg/dL	0.1–1.2 mg/dL
Creatine kinase	208 U/L	26–192 U/L

**Figure 1:**
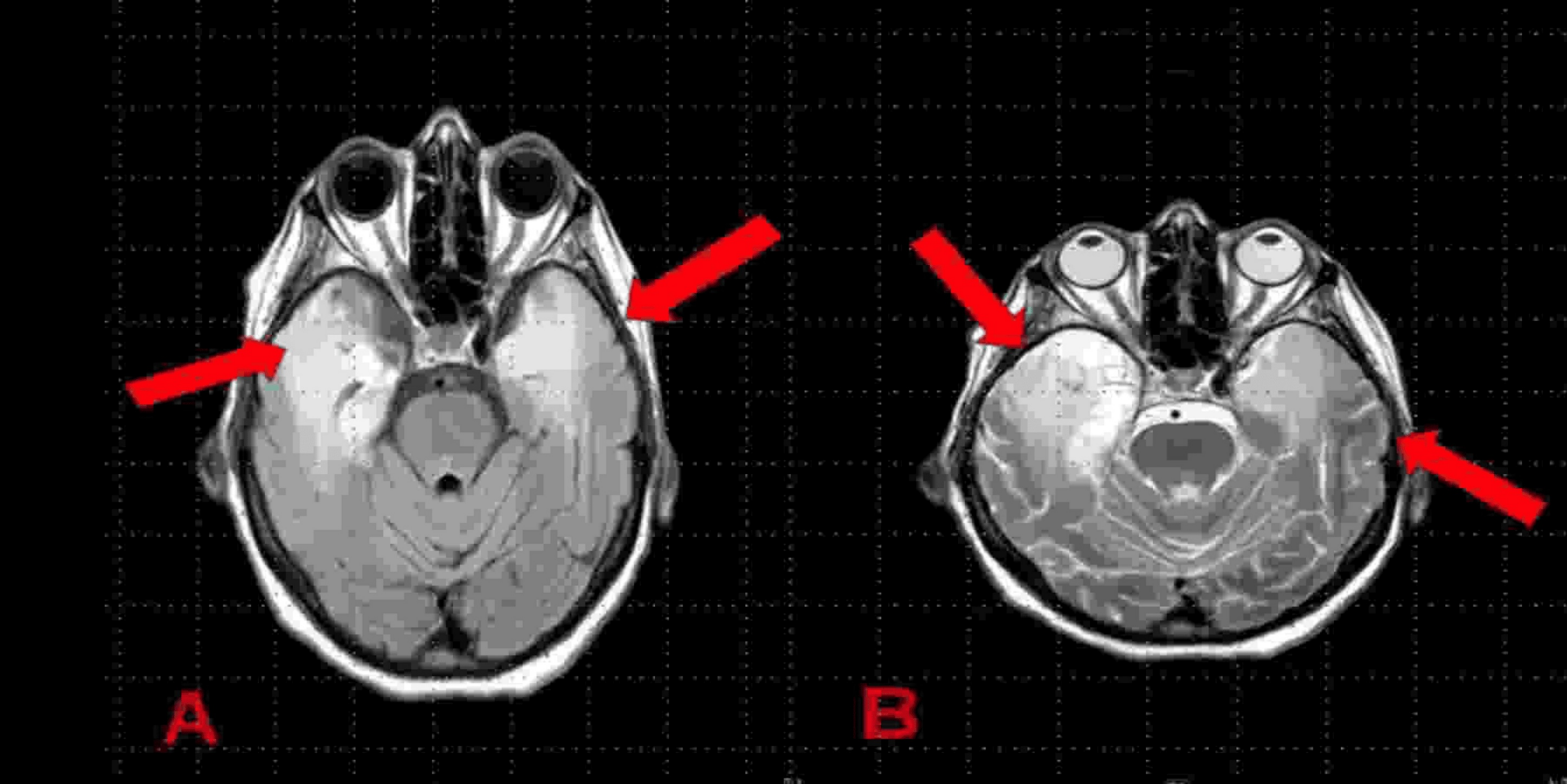
Brain MRI showing diffuse abnormal T2/fluid-attenuated inversion recovery hyperintense signal in the bilateral temporal lobes (A) and bilateral hippocampi (A, B) (red arrows). The MRI was taken on day six after the patient presented with worsening symptoms of confusion and a Glasgow Coma Scale score <5.

On day two, the patient presented with a sudden decline in mentation and developed a fever of 102.4°F with tachycardia. Labs were significant for mild hyponatremia at 131 mmol/L. Magnetic resonance imaging (MRI) of the brain was without any findings suggesting acute intracranial infection or process. Chest X-ray revealed bilateral scattered patchy airspace opacities, and urinalysis demonstrated bacteriuria and pyuria, followed by negative urine culture, in the setting of no reported urinary symptoms, likely due to asymptomatic bacteriuria versus sample contamination. The patient was started on intravenous (IV) fluids, azithromycin, and ceftriaxone for suspected underlying infection. On day four, CT of the chest revealed a small pleural effusion with atelectatic changes. On day six, the patient was still febrile with a progression of respiratory distress, so she was transferred to the intensive care unit (ICU), where she underwent endotracheal intubation for airway protection. She was lethargic, disoriented, and unresponsive with a Glasgow Coma Scale score of 5. MRI of the brain revealed abnormalities of the right temporal lobe, hippocampus, right insular cortex, and right inferior frontal gyrus suggestive of herpes simplex encephalitis (see Figure 1). A spinal tap was performed, opening pressure was normal with lymphocytic pleocytosis, WBC count of approximately 0.15 × 10^3^ cells/µL, elevated protein level of 190 mg/dL, and a glucose level of 54 mg/dL (see Table 3). Cerebrospinal fluid (CSF) polymerase chain reaction (PCR) for HSV-1 was positive, and HSV-2 IgG antibodies were present.

**Table 3:** Cerebrospinal fluid analysis.

Cerebrospinal fluid analysis	Results	Normal values
Appearance	Colorless and clear	Colorless and clear
White blood cells	0.153 × 10^3^ cells/µL	0–5 × 10^3^ cells/µL
Red blood cells	61 cells/µL	<1 cell/µL
Glucose	54 mg/dL	50–80 mg/dL
Protein	190 mg/dL	15–60 mg/dL
Herpes simplex virus 1 DNA polymerase chain reaction	Detected	Negative

**Figure 2:**
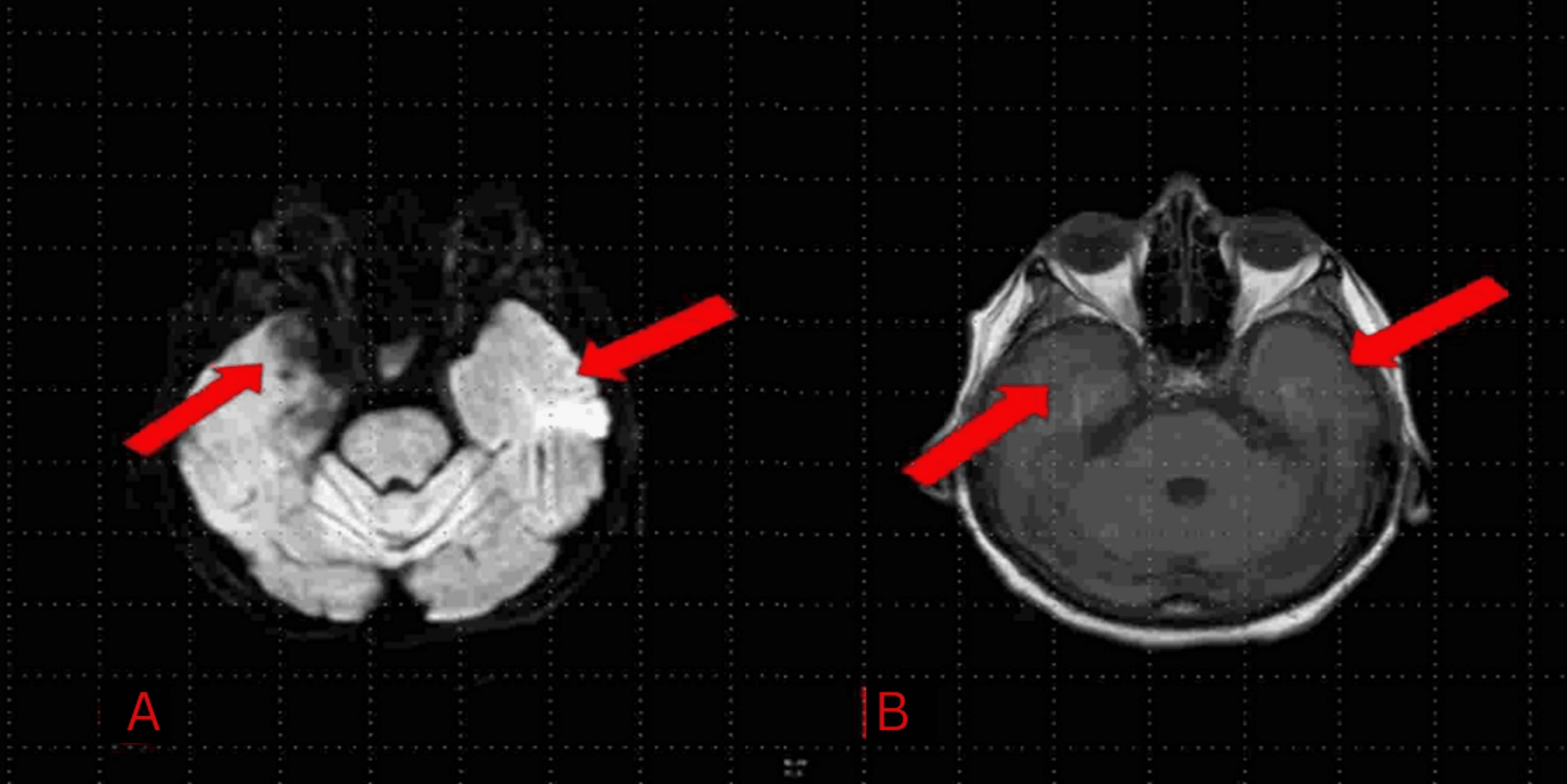
Focal subacute to chronic blood products in the right temporal lobe (A, B) consistent with inflammation and necrosis due to herpes simplex virus type 1 encephalitis (red arrows). The MRI was taken on day 18 to evaluate for improvements or worsening of the initial findings of the MRI on day 6 after treatment with acyclovir and ganciclovir.

In addition to broad-spectrum antibiotics, the patient was started on empiric antiviral therapy. She completed acyclovir for 14 days and was transitioned to ganciclovir for seven days due to a hospital-wide shortage of acyclovir. On day seven, her fever started to improve, and she was started on seizure prophylaxis with lacosamide (Vimpat), levetiracetam (Keppra), and a midazolam drip. The patient was initially extubated on day 12 but re-intubated again on day 14 due to airway protection caused by altered mental status with non-convulsive status epilepticus. Overnight on days 15-16, continuous electroencephalogram (EEG) revealed status epilepticus, and the patient received valproic acid with resolution of seizures. On day 18, a brain MRI indicated that the patient had slightly worsening inflammation of the left and right temporal lobes as compared to her initial MRI (see Figure 2).

**Figure 3:**
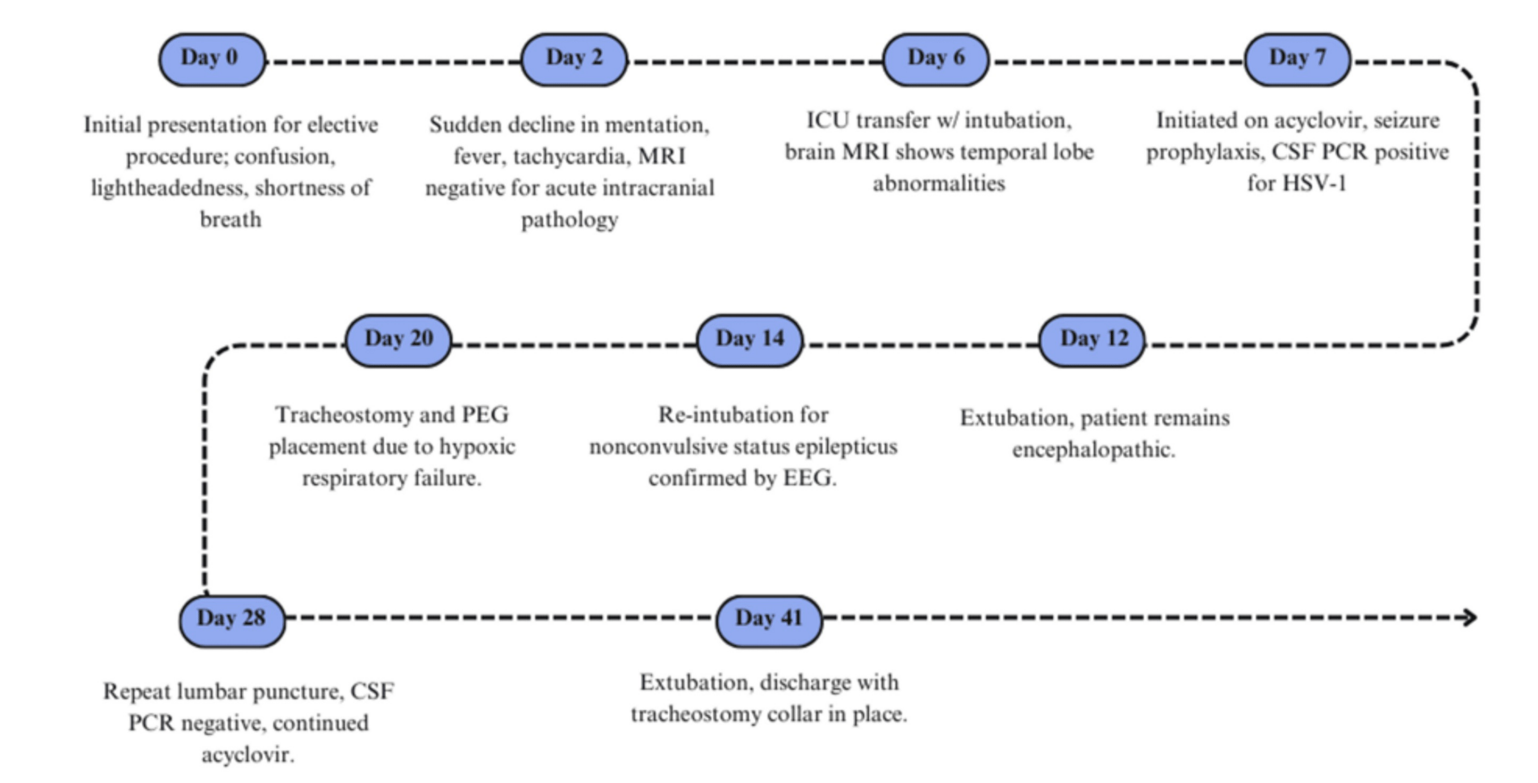
Clinical timeline of the patient’s progression with HSV-1 encephalitis. ICU: intensive care unit; CSF: cerebrospinal fluid; PCR: polymerase chain reaction; PEG: percutaneous endoscopic gastrostomy; EEG: electroencephalogram; HSV-1: herpes simplex virus type 1

On hospital day 20, the patient underwent tracheostomy and percutaneous endoscopic gastrostomy placement for ongoing hypoxic respiratory failure. A repeat lumbar puncture was performed on day 28, which showed an opening pressure of 22 mmHg, and CSF PCR for HSV-I was negative. The patient was prescribed valacyclovir for post-exposure prophylaxis to complete for three to six months. On day 35, the EEG indicated moderate-to-severe diffuse cerebral dysfunction. On day 37, the patient’s family agreed to transfer to hospice care in the setting of significant functional, nutritional, and cognitive impairment. The patient’s neurological status remained unchanged during her ICU course. The patient’s neurological status remained unchanged from baseline during her ICU course; she remained encephalopathic, intermittently able to open her eyes, and unable to withdraw to noxious stimuli on any extremity. She was ultimately extubated on day 41 and discharged with a tracheostomy collar in place. The patient’s clinical course, including key interventions and outcomes, is summarized in the timeline in Figure 3.

## Discussion

Two mechanisms of HSV-1 encephalitis have been proposed, either through reactivation of a latent infection or through a primary central nervous system (CNS) infection. It is believed that herpes simplex encephalitis occurs by retrograde transport via the olfactory and trigeminal nerves into the CNS with preferential targeting of the mesiotemporal and orbitofrontal lobes [1,2,5]. The profound neurological deficits can be explained by the predilection for the mesial temporal lobes, which are responsible for cognitive functions. Structures found in the temporal area of the brain, such as the amygdala, hippocampus, insula, and cingulate gyrus, are mainly affected during infection and are responsible for important cognitive functions, which can be permanently damaged postinfection [3,6]. The infection results in necrosis and hemorrhage due to viral cytolysis of neurons, glial cells, and endothelial cells [5]. The inflammatory response induced by activated leukocytes is at the base of the neurological destruction and sequelae [7,8]. Results of these processes include acute complications, such as seizures, cerebral edema, and secondary bacterial infections, which can cause pneumonia or sepsis [5]. Other complications of infection include cerebrovascular incidents such as stroke or intraparenchymal hemorrhages [9]. Early detection and treatment of herpes simplex encephalitis can help reduce the risk of these complications and prolonged hospitalization of patients.

The first-line imaging modality for HSV-1 encephalitis is MRI of the head with and without contrast [1]. The typical findings seen on MRI include asymmetric hyperintense lesions localized to the frontotemporal lobes and signs of hemorrhage and edema [10,11]. These findings were seen in our patient, as shown in Figure 1. CT of the head can also be utilized to visualize signs of infection, corresponding with hypointense lesions in an asymmetric distribution bilaterally in the frontotemporal lobes [11]. CSF analysis and detection of viral DNA via PCR are also an important step in the diagnostic evaluation of patients suspected of having HSV-1 encephalitis. CSF analysis will typically indicate lymphocytic pleocytosis with likely elevations in WBC counts, protein, and possible decrease in glucose concentration [1,2]. A systematic review and meta-analysis of PCR testing for HSV-1 in the CSF found that the sensitivity was greater than 97%, concluding high accuracy for detecting HSV-1 in the CSF [12]. However, multiple studies have shown that early infection by HSV-1 can result in false negatives on PCR testing [12]. The combined use of CSF analysis, PCR testing, and brain imaging was performed multiple times on our patient, both for diagnostic evaluation and to gauge treatment response over the course of her hospital stay.

EEG can also be used to aid in the diagnosis of HSV-1 encephalitis. Typical findings include epileptogenic features in the medial temporal and hippocampal brain regions [2]. Specifically, sharp-and-slow complexes that originate in the temporal regions at two to three-second intervals that are characteristically recurrent and uniform have been described [2,13]. One study of encephalitis cases determined that patients with these features on EEG were more likely to have encephalitis due to HSV-1 infection rather than an alternate cause [13]. These features can be seen within a time interval of 2-15 days after infection and before the appearance of hypointense lesions seen on CT imaging [1,2]. In our patient, EEG showed cortical irritability in the bilateral frontotemporal lobes with a high risk of focal onset seizures, for which she was subsequently started on seizure prophylaxis.

The first-line treatment option for patients with HSV-1 encephalitis is IV acyclovir. Multiple studies have shown the effectiveness of acyclovir treatment with a reduction in mortality from 70% in untreated patients to 20% in patients started on acyclovir early on in the infection [1,14,15]. A literature review of three case reports detailing the disease management of HSV-1 encephalitis found that there are potential benefits in individualizing acyclovir treatment for patients based on CSF analysis and HSV-1 quantification [16]. Discontinuing acyclovir treatment before complete viral eradication can lead to significant neurological sequelae. Assessing a patient’s viral load may help determine the appropriate duration of antiviral therapy to optimize outcomes [17]. The Infectious Disease Society of America recommends IV acyclovir 10 mg/kg every eight hours in an immunocompetent adult patient [15]. If IV acyclovir is not available, the second-line treatment is ganciclovir [2]. In our case, the patient initially started antiviral therapy with acyclovir but was transitioned to ganciclovir due to a hospital-wide shortage of acyclovir, ensuring uninterrupted antiviral treatment.

Current guidelines recommend that physicians have a low threshold for suspecting HSV-1 encephalitis in patients presenting with common symptoms. All patients with suspected herpes simplex encephalitis, pending results of diagnostic studies, or confirmed cases must be immediately started on IV acyclovir as soon as possible [15]. Typically, the patient will complete a 14-21-day course on antiviral therapy [1]. In our patient, she was started empirically on acyclovir after her MRI, which was highly suspicious for herpes simplex encephalitis. A large retrospective study found that a delay of more than 48 hours in starting acyclovir was associated with poor outcomes. This delay contributed to the deaths of 13 (15%) patients and severe disability in 17 (20%) patients among 93 adult cases [17]. Based on our literature review of other HSV-1 encephalitis case reports, the implementation of IV acyclovir as soon as possible resulted in faster and improved outcomes [18-20]. This includes a case in which a patient had HSV-1 encephalitis with multi-organ involvement after oral corticosteroid treatment, a patient with HSV-1 relapse after epilepsy surgery, and a case of subacute HSV-1 encephalitis [18-20].

## Conclusions

We report a case of HSV-1 encephalitis in an elderly patient presenting with an acute onset of symptoms before an elective outpatient procedure. This case underscores the rarity of the condition presenting unexpectedly in a patient undergoing preoperative evaluation for an elective procedure. The patient’s rapid progression to status epilepticus and subsequent neurological decline highlights the importance of considering HSV-1 encephalitis in atypical presentations. HSV-1 encephalitis is a devastating condition associated with significant morbidity and mortality, and timely recognition and intervention are crucial to improving patient outcomes, particularly in cases with unusual clinical onset.
